# Naphtha in Pulmonary Consumption

**Published:** 1844-06

**Authors:** 


*Naphtha in Pulmonary Consumption.* Case I.—July 14, 1843. A. N., ætat. 32, has had cough and expectoration of opake yellow matter for some months, with emaciation. Was able to pursue her occupation, that of a dress-maker, until the spring of the present year, when her debility was much increased. Percussion dull over the left side throughout its whole extent; respiration cavernous in the same side as low as the third rib; pectoriloquy perfect; percussion natural on the right side; respiration bronchial; no râle. To take ten drops of naphtha three times in a day. After taking it a few days, she "thought it did her cough good." She continued the naphtha without the least apparent benefit until within a few days of her death, which took place September 11. Whilst taking the naphtha, the night perspirations were much increased.

Case II.—July 13, 1843. S. M., ætat. 20; four months ago, being in service in London, was attacked with cough, accompanied with much debility and languor; returned home a fortnight since. Countenance unhealthy; expectorates globular masses, of a greenish white color; pulse rapid; perspires much at night. The subclavicular spaces on either side are much flattened, and preternaturally sonorous on percussion; under the right clavicle the inspiration is accompanied with a single moist crapitation; pectoriloquy perfect. On the left side the respiration is rough and grating, and a puff is heard to strike at the bottom of the cylinder at each effort at articulation; sounds of the heart diffuse. After taking ten drops of naphtha three times daily for three weeks, the expectoration somewhat lessened, but again increased in a few days; the dose was augmented to fifteen drops. Colliquative diarrhœa set in, which was with difficulty checked by opium. As she considered the naphtha aggravated the diarrhœa, she was ordered to omit it for a few days, but as little alteration was observed in the frequency of the motions, she resumed it; becoming, however, rapidly worse, she finally abandoned it, after having taken it about a month. She died December 3.

Case III.—August 18, 1843. Mrs. S., ætat. 30, had been affected during the last winter with cough and shortness of breath, which confined her to her house. During the summer her health improved, so that she was able to take walking exercise in the open air. Towards autumn her health again declined; she was troubled with night perspirations, cough and expectoration; catamenia suppressed. Percussion elicited a dull sound under the right clavicle, in which situation a small crepitation is heard at the end of each inspiration. On the left side the inspiration is broken. Took the naphtha, in doses of fifteen drops, for nearly a month, when it was abandoned at her own request. During this time the symptoms progressed from bad to worse without any mitigation. She is now entirely confined to her bed, and her death is daily expected.

Case IV.—July 20, 1843. O. J., ætat. 24, family consumptive; had dry cough at the beginning of the same year, which did not much interfere with his health until the middle of May, when he was attacked with hæmoptysis, which lasted more or less for two days, since which time he remained much debilitated. Face pallid and thin; coughs much, and expectorates mucus with yellowish masses suspended in it; is capable of walking about, and can lie on either side; pulse 94; perspires at night; percussion rather dull in the left subclavicular region, as far as the third rib; respiratory murmur in this situation feeble, and occasionally broken; a faint crepitation may sometimes be detected at the termination of each expiration; much vocal resonance; percussion under the line of the right clavicle slightly dull; respiration over the whole of this side puerile; no râle. He commenced taking naphtha three times daily, in doses of ten drops, which were gradually increased to twenty. The only effects observed were that it produced severe griping, aggravated the diarrhœa, and, while under its use, perspirations were most profuse. To control its tendency to increase the diarrhœa, a few drops of laudanum were given in conjunction with the naphtha.

With much perseverance this patient persisted in its use, although to him a very disagreeable medicine, until the 1st of September, a period of six weeks, when he could not be persuaded to go on with it longer. During the time he was taking it, the cough, expectoration, hectic fever, and emaciation increased rapidly; and stethoscopic examination discovered progressive softening and disorganization of the upper part of the left lung. He died in October.

Case V.—August 18, 1843. Mrs. S., grocer, of stout, robust make, has for several winters been troubled with cough and expectoration. In the summer of the present year spat blood for two or three days, since which has had much expectoration of muco-purulent matter, frequently tinged with blood; has lost flesh, but is still capable of partially serving in her shop. Percussion dull under the left subclavicular space, which is considerably flattened, and in which situation a moist crepitous râle is heard. To take 20 drops of naphtha three times a day.

September 28. Is evidently weaker; cough violent; expectoration copious, and much dyspnœa on exertion. States that the only effect which she can ascribe to the drops is the induction of some degree of headache after taking them.

After persevering in the use of naphtha for nearly two months, without benefit, she discontinued it, at which time the crepitating râle had given place to a strong and extensive gargouillement. Her condition is now much the same as when she discontinued the use of the naphtha.

Case VI.—Aug. 12, 1843. Mrs. S., æt. 29, has had cough since last Christmas, but without much disturbance of the general health, until about three months ago; she then experienced debility and night perspirations. Her present symptoms are as above detailed; expectoration sparing; pulse very rapid; catamenia suppressed for two months; percussion very dull below the left clavicle, inspiration here terminating in a slight mucous râle; expiration prolonged, rough, and blowing; pectoriloquy distinct. After taking the naphtha in doses of twenty drops, for a month, without any benefit, and with rapid advancement of the disease, she discontinued it.

Case VII.—October 1, 1843, J. F., ætat. 32, states that he has had cough for some weeks; expectorated recently about a teaspoonful of blood; suffers from shortness of breath, but is tolerably robust in appearance; on percussion a trace of dullness is perceived immediately under the right clavicle; the inspiration here is feeble; expiration broken. After he had taken twenty drops of naphtha three times a day, for a fortnight, with little or no relief to his cough or breathing, I did not feel justified, after the naphtha had hitherto entirely failed in my hands, in omitting any other means which I thought might be beneficial to the patient. I therefore kept up counter-irritation under each clavicle for several days; at the same time he continued the use of the naphtha. Under this plan his health improved, his cough moderated, and his breathing became less difficult, and for the last three weeks has been able to fulfil the duties of his avocation, that of a farm-laborer. He ascribes his recovery to the repeated blisters. Whether these, or the naphtha, be entitled to the credit, I shall forbear to express my opinion.

From an attentive consideration of the cases which have been imperfectly detailed above, I cannot hesitate to state that, at present, I am sceptical as to any beneficial influence exercised by naphtha in cases of true tubercular phthisis. At the same time I shall not fail to give it a more extended trial, as opportunity offers.—*London Lancet*, for January.

